# Nitrogen Immobilization in Organic Media: A Double-Edged Sword Affecting the Utilization of Green Waste as Growing Media

**DOI:** 10.3390/plants15091298

**Published:** 2026-04-23

**Authors:** Ruohan Li, Wenzhong Cui, Min Zhang, Zhiyong Qi, Wanlai Zhou

**Affiliations:** Institute of Urban Agriculture, Chinese Academy of Agricultural Sciences, Chengdu 610213, China; liruohan000120@163.com (R.L.); 17784240198@163.com (W.C.); zhangmin10062023@163.com (M.Z.)

**Keywords:** organic residues, soilless substrates, Biotic N immobilization, abiotic N immobilization

## Abstract

This review proposes a “phenomenon–mechanism–regulation” framework for understanding nitrogen immobilization during the conversion of green waste into growing media. Nitrogen immobilization acts as a double-edged sword: intense short-term immobilization, typically occurring within the first 1–2 weeks after substrate establishment, can rapidly deplete mineral nitrogen and induce plant nitrogen deficiency, whereas the immobilized nitrogen is subsequently incorporated into microbial biomass and lignin-associated organic pools, forming a slow-release reservoir that enhances nitrogen retention and reduces leaching losses. Owing to its extremely high C/N ratio (often >100) and the coexistence of labile carbon fractions and recalcitrant compounds (e.g., lignin and phenolics), green waste exhibits substantially stronger immobilization potential than conventional media. Empirical evidence indicates that nitrogen immobilization can reach 10–115 mg N·L^−1^ within a few days in wood-derived substrates, and additional fertilization of up to 100 mg N·L^−1^ may be required to maintain crop growth. Mechanistically, nitrogen immobilization is governed by the coupling of microbial assimilation—driven by stoichiometric C/N imbalance (typically triggered when C/N > 20–25)—and abiotic chemical fixation, including reactions between NH_4_^+^/NO_2_^−^ and lignin-derived phenolics forming stable organic nitrogen compounds. The relative dominance of these pathways is jointly regulated by carbon quality, nitrogen form, and pH. Based on these mechanisms, regulatory strategies are summarized at multiple scales, including feedstock pretreatment to reduce labile carbon availability, substrate formulation to optimize C/N balance, and model-assisted intelligent fertigation to synchronize nitrogen supply with crop demand. Overall, this study provides a theoretical basis for improving green waste valorization and promoting sustainable horticultural production.

## 1. Introduction

Growing media serve as the physical carrier and nutrient interface in soilless culture systems and therefore strongly influence the rhizosphere environment and crop productivity [1]. Peat has long been the preferred medium because of its favorable physicochemical properties; however, its non-renewability and the ecological damage caused by its extraction have made the search for sustainable, locally available alternatives an urgent priority in horticulture [2,3,4,5].

Among various candidates, green waste is considered a promising peat alternative because it is abundant, renewable, and relatively inexpensive. Globally, the generation of municipal solid waste continues to increase and is projected to rise from approximately 2.1 billion t in 2023 to 3.8 billion t by 2050, indicating that the management pressure associated with organic wastes, including green waste, will further intensify [6]. In China, green waste has become the second largest category of organic solid waste in urban areas after municipal household waste, with an annual generation of approximately 40–70 million t. Moreover, its production has increased rapidly, rising from 10.7134 million t in 2003 to 60.9705 million t in 2022, and is projected to reach 83.35–91.48 million t by 2030 [7]. In contrast, countries in Europe and the United States incorporated garden waste into systems for the separate collection and resource utilization of biowaste at an earlier stage. For example, in the United States, yard trimmings amounted to approximately 35.4 million t in 2018, accounting for 12.1% of total municipal solid waste. In the European Union, municipal waste generation reached 511 kg per capita in 2023, of which 48% was recycled through material recovery, composting, and related pathways [8,9]. However, despite this vast resource potential and decades of exploration, the high-value utilization of green waste in professional horticulture remains disproportionately low. This discrepancy highlights a persistent gap between the theoretical availability of biomass and the practical stability of substrate performance.

Predominantly composed of plant fibers, green waste can be processed into growing media with excellent water-holding capacity and aeration performance, while providing essential nutrients such as N, P, and K [2,10,11]. Its local availability may also reduce the carbon footprint of substrate production and transport, aligning with the principles of circular agriculture. However, its high C/N ratio often induces nitrogen immobilization, which remains a critical bottleneck restricting the large-scale application of green waste as growing media [12,13,14]. While nitrogen immobilization is traditionally viewed as a simple biological competition for nutrients, emerging evidence suggests a more complex interplay between microbial consumption and abiotic chemical immobilization. Nevertheless, the relative contributions of these biotic and abiotic pathways, particularly in high-lignin substrates, remain a subject of ongoing debate and lack quantitative clarity.

**Figure 1:**
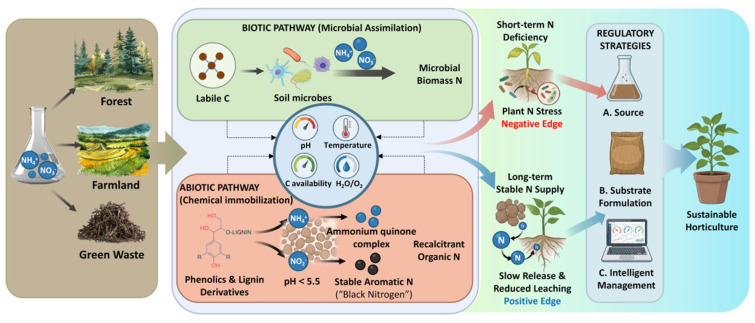
Nitrogen immobilization in organic media: mechanisms, effects, and regulatory strategies.

This process (nitrogen immobilization) has a dual role in green-waste-based media. In the short term, strong immobilization can induce nitrogen deficiency in plants. In the longer term, however, the immobilized nitrogen may contribute to a stable organic nitrogen pool that is gradually remineralized, thereby supporting sustained nutrient supply and reducing the risks of nitrate leaching and ammonia volatilization. Current research, however, tends to focus heavily on the detrimental short-term effects, often overlooking the long-term ecological benefits and the potential to steer this process via precise management. Because nitrogen supply, transformation, and retention are central to agricultural productivity and environmental sustainability [15], a comprehensive re-evaluation of nitrogen immobilization in green-waste-based media is essential. However, current knowledge is fragmented: microbial immobilization has been extensively studied, whereas abiotic pathways, particularly those involving lignin-derived compounds, remain poorly characterized, and their relative contributions are rarely integrated into existing frameworks. Moreover, most current studies are case-specific and lack a unified conceptual framework linking mechanisms, controlling factors, and management strategies. This review therefore examines its biochemical mechanisms and major controlling factors and proposes a regulatory framework from feedstock pretreatment to cultivation management, with the aim of supporting the conversion of green waste into a more efficient and manageable horticultural resource (Figure 1).

## 2. Nitrogen Immobilization in Organic Media

### 2.1. Nitrogen Immobilization in Natural Soil

Nitrogen immobilization is a fundamental biogeochemical process in natural soils. By converting reactive inorganic N—primarily ammonium (NH_4_^+^) and nitrate (NO_3_^−^)—into organic forms, this process plays a key role in regulating nitrogen retention, primary productivity, and environmental risk in terrestrial ecosystems. The intensity of this process exhibits significant ecosystem dependency, and its quantitative characteristics have been systematically characterized on a global scale. A comprehensive meta-analysis of 398 global ^15^N isotope dilution and tracer experiments revealed average immobilization rates of 1.93 ± 0.31 and 8.17 ± 0.94 mg N kg^−1^·d^−1^ for NO_3_^−^-N and NH_4_^+^-N, respectively [16]. These findings globally validate that most natural soils possess a higher affinity and immobilization capacity for NH_4_^+^. This preference dictates the primary pathway for the flux of inorganic nitrogen into organic pools. This mechanism serves as the cornerstone for nitrogen retention and buffering capacity within ecosystems.

In natural forest ecosystems, the continuous input of litter with high C/N ratios provides abundant carbon substrate and energy for microbes, thereby driving intensive microbial nitrogen immobilization. Global meta-analyses indicate that microbial nitrogen immobilization rates in forest soils reach 8.10 mg N kg^−1^·d^−1^, significantly exceeding the average gross N mineralization rate of 6.65 mg N kg^−1^·d^−1^. Furthermore, these rates exhibit significant positive correlations with soil organic carbon, microbial biomass carbon, and the C/N ratio [17].

In contrast, nitrogen immobilization in agricultural ecosystems is strongly influenced by anthropogenic management, exhibiting distinct intensities, mechanisms, and dynamics compared with forest soils dominated by natural litter inputs. Generally, intensive agricultural practices tend to suppress the soil’s nitrogen immobilization capacity [16]. A comparative study demonstrated that NH_4_^+^ immobilization in forest soils can reach 6.67 mg N kg^−1^·d^−1^, whereas it drops to only 0.34 mg N kg^−1^·d^−1^ in adjacent croplands. This disparity underscores that land-use conversion from forest to cropland significantly weakens the soil’s internal nitrogen retention capacity [18].

### 2.2. Nitrogen Immobilization in Growing Media

In substrate-based systems, the rhizosphere microenvironment—characterized by optimal aeration and water-holding capacity—promotes rapid microbial proliferation and metabolic activity, thereby intensifying the competitive uptake and temporary immobilization of exogenous inorganic nitrogen. Simultaneously, due to the confined rhizosphere volume, limited physicochemical buffering capacity, and small nutrient reservoir of growing media, any short-term fluctuations in nitrogen availability are rapidly transmitted to the root system. Consequently, the impact of nitrogen immobilization on crop growth is more immediate and pronounced in soilless growing media than in traditional soil-based systems.

Nitrogen immobilization in growing media has been recognized since the 1990s and occurs mainly in organic substrates [19,20,21]. Its intensity depends largely on feedstock composition and maturity. Peat generally shows little immobilization because it is highly humified [22,23]. In contrast, coconut coir, typically characterized by a C/N ratio of 80–100 and high lignin content (approx. 35–45%), exhibits moderate immobilization. Based on its reported Nitrogen Drawdown Index (NDI), this may necessitate a 15–20% compensation in initial nitrogen loading compared to peat to ensure optimal seedling nitrogen uptake [24,25]. Research indicates that these woody substrates can necessitate an additional 50–100 mg·L^−1^ of supplemental inorganic nitrogen to compensate for rapid microbial assimilation. Furthermore, based on established diagnostic protocols, fresh woody materials often yield a Nitrogen Immobilization Index (NII) exceeding the critical threshold of 100 mg N·L^−1^ substrate, whereas stabilized or composted materials typically remain below 50 mg N·L^−1^, highlighting the significant impact of material maturity on nitrogen dynamics [26,27].

Green waste typically possesses extremely high C/N ratios, which directly triggers intensive microbial nitrogen immobilization [19,20,23]. Quantitative assessments reveal that the nitrogen immobilization intensity in these growing media far exceeds that of mineral soils or conventional organic media: For instance, fresh pine wood growing media exhibit an nitrogen immobilization capacity of 10–20 mg N·L^−1^ within just four days, whereas specific wood fiber growing media can reach as high as 115 mg N·L^−1^ [22,28]. This intensive immobilization leads to a severe depletion of plant-available nitrogen. Studies indicate that tomato plants grown in pure wood fiber require an additional N supplementation of up to 100 mg N·L^−1^ to achieve biomass levels comparable to those grown in peat [14].

### 2.3. The Dual Role of Nitrogen Immobilization

Nitrogen immobilization is inherently dual-natured, and its effects are context-dependent, reflecting a trade-off between short-term limitation of plant-available N and long-term formation of a N reservoir. In natural or semi-natural ecosystems such as forests and croplands, nitrogen immobilization is generally regarded as an ecological buffering mechanism that helps reduce nitrogen losses and maintain nutrient cycling. However, in growing substrates dominated by garden waste with a high C/N ratio, this process can rapidly immobilize inorganic nitrogen in the short term, thereby restricting the immediate nutrient availability for crops.

In these ecosystems, nitrogen immobilization functions as a key ecological buffering mechanism. Under increasing atmospheric N deposition, microbial immobilization rapidly sequesters both exogenous and mineralized inorganic N, thereby delaying its transfer into leaching pathways [29]. Especially in carbon-rich systems, this process serves as a dominant mechanism for regulating the balance between N retention and loss in N-saturated ecosystems [30]. Agricultural studies have demonstrated that incorporating organic amendments with high C/N ratios (e.g., wheat or rice straw) promotes microbial immobilization and physicochemical adsorption, which sequesters excess mineral N in the short term while reducing nitrate leaching and N_2_O emissions [31,32,33]. This mechanism remains effective under both flooded and arid conditions. Furthermore, it acts synergistically with chemical fertilizers to facilitate slow-release nitrogen supply, thereby improving nitrogen use efficiency [34,35].

In contrast, the negative impacts of nitrogen immobilization are significantly amplified in cultivation growing media dominated by high C/N ratio organic materials, such as green waste. Intense microbial N assimilation is typically initiated within days of transplanting and peaks around two weeks [22]. This process leads to a rapid decline in mineral N concentration in the substrate solution. This induces severe N deficiency during critical early growth stages, which manifests as growth retardation and foliar chlorosis and further impairs the plant’s photosynthetic physiology [36]. During this phase, the organic substrate may transition from a potential nutrient source to a nutrient competitor for plants. However, the latent positive effects of nitrogen immobilization persist: the sequestered nitrogen is not permanently lost but is instead incorporated into microbial biomass and necromass. Structural organic components such as lignin may also sequester nitrogen through chemical bonding. Together, these processes form an organic N pool that gradually releases nutrients through mineralization over subsequent weeks or months [37]. Furthermore, intensive microbial nitrogen immobilization alters rhizosphere chemical signaling and nutrient gradients, potentially stimulating plant roots to excrete organic acids as a stress response. This may indirectly influence the bioavailability of elements such as phosphorus (P) and iron (Fe) and selectively enrich specific rhizosphere microbial communities [38]. Such processes may foster symbiotic relationships between plants and rhizosphere microbes, thereby enhancing abiotic stress tolerance and the nutrient acquisition capacity of plants.

## 3. Mechanisms of Nitrogen Immobilization

Nitrogen immobilization in organic media is governed by two fundamentally different but interacting mechanisms: microbial assimilation and abiotic chemical immobilization.

### 3.1. Microbial Nitrogen Immobilization

Microbial nitrogen immobilization is the most widely recognized mechanism driving N fixation in organic media, fundamentally stemming from microbial assimilation based on stoichiometric requirements [39]. Owing to their higher specific surface area and more rapid growth rates compared to plant roots, microbes typically possess a competitive advantage in the acquisition of available nitrogen [40]. In high-C/N media, the rapid proliferation of microorganisms triggers the rapid uptake of inorganic NH_4_^+^ and NO_3_^−^ from the substrate solution to maintain a stable cellular C/N ratio (typically 5 to 10), thereby causing local nitrogen deficiency. Empirical studies demonstrate the high efficiency of this process. For instance, in acidic spruce forest soils, 50–60% of exogenous N is rapidly incorporated into microbial biomass within 48 h [30]. In agricultural systems, wheat straw facilitates the rapid immobilization of excess nitrogen, storing it as microbial biomass nitrogen (MBN), with rates reaching 42 kg N ha^−1^ within 7 days [31]. The sequestered nitrogen is primarily synthesized into intracellular components, including peptides, proteins, and nucleic acids [41]. Microbial nitrogen immobilization is mediated by a functional consortium of bacteria, fungi, and actinomycetes rather than by a single group. These microorganisms cooperate in lignocellulose degradation and nitrogen transformation, thereby sustaining nitrogen immobilization across decomposition stages [42,43,44,45]. This biotic process is characterized by its rapid onset and potential reversibility; as carbon sources are exhausted, the immobilized nitrogen is eventually returned to the plant-available pool through microbial turnover and remineralization.

### 3.2. Abiotic Nitrogen Immobilization

Beyond microbial nitrogen immobilization, abiotic chemical immobilization represents another crucial pathway for nitrogen sequestration in organic media, particularly in acidic environments with high organic matter content. It proceeds through direct reactions between inorganic N and reactive organic functional groups and can occur rapidly, with NH_4_^+^, NO_3_^−^, and NO_2_^−^ all participating under suitable chemical conditions [46,47].

Currently, our understanding of the mechanisms underlying abiotic nitrogen immobilization in organic materials remains quite limited. Both NH_4_^+^ and NO_2_^−^ exhibit high chemical reactivity toward various soil organic compounds (e.g., humic and fulvic acid analogs, as well as lignin derivatives), thereby driving abiotic nitrogen immobilization within the soil matrix [48]. Specifically, NH_3_ can undergo condensation reactions with activated phenols or quinone rings to form nitrogenous humates [49,50]. NH_4_^+^ may react chemically with lignin-derived phenols, facilitating the abiotic sequestration of nitrogen in the soil [51]. Additionally, NO_2_^−^ reacts with lignin or its derivatives (e.g., phenolic compounds) to form recalcitrant organic N conjugates [52,53]. Using ^15^N labeling, researchers found that NO_2_^−^ primarily reacts abiotically with fulvic acids, sequestering ~6% of applied NO_2_^−^-N into stable forms such as amides and pyrrolic “black nitrogen” [54]. Furthermore, NO_3_^−^ can be converted into organic nitrogen species under specific conditions via the so-called “ferrous wheel mechanism” [55]. Lignin likely plays a pivotal role in abiotic nitrogen immobilization [56,57]. Collectively, abiotic nitrogen immobilization is characterized by rapid chemical reactions and the formation of stable, low-turnover organic nitrogen pools, particularly under conditions rich in reactive phenolics and acidic environments. Nevertheless, research on nitrogen immobilization has predominantly focused on organic-rich soils within forests, grasslands, oceans, and agricultural lands [58]. Consequently, for growing media such as green waste—which are uniquely characterized by high concentrations of lignin and polyphenols—the intensity, specific pathways, and relative contributions of abiotic nitrogen immobilization remain poorly understood. This constitutes a critical knowledge gap for the valorization and sustainable application of green waste as growing media.

### 3.3. Dominant Factors in Nitrogen Immobilization Pathways

The relative contributions of nitrogen immobilization pathways—both biotic and abiotic—are primarily determined by the C/N stoichiometric status of the system, with carbon (C) sources and N availability acting as co-regulators [59]. When organic matter is abundant in labile carbon (e.g., sugars, organic acids), microbial metabolism is strongly stimulated, thereby rendering biotic immobilization the dominant pathway. Conversely, if the organic matter is dominated by recalcitrant components (e.g., lignin-derived phenols), abiotic immobilization through chemical reaction pathways is favored. Nitrogen availability further modulates this balance: during the N-limitation phase, microbial assimilation governs the nitrogen immobilization process. As N sufficiency shifts microbial metabolism toward carbon limitation, the relative contribution of abiotic processes increases significantly, even becoming predominant under N-rich conditions [50].

## 4. Key Factors Influencing Nitrogen Immobilization in Growing Media

### 4.1. Material Composition

The substrate biochemical profile acts as the primary determinant; specifically, the balance between labile carbohydrates and recalcitrant lignin determines whether immobilization is dominated by rapid microbial uptake or stubborn chemical sequestration. Among various parameters, the carbon-to-nitrogen (C/N) ratio serves as the primary empirical indicator for assessing nitrogen immobilization potential [60,61]. It is generally accepted that when the C/N ratio exceeds 20–25, microbial growth becomes N-limited, prompting the assimilation of exogenous or mineralized inorganic N, which leads to net nitrogen immobilization [62]. However, as a macroscopic stoichiometric index, the C/N ratio possesses inherent limitations in its predictive capacity [63]. The bioavailability of carbon sources often predicts the occurrence and intensity of nitrogen immobilization more accurately than total carbon content [58]. If a high C/N ratio originates from recalcitrant components such as lignin and cellulose, microbial metabolism remains sluggish, potentially delaying or attenuating nitrogen immobilization. Conversely, materials rich in labile carbon sources (e.g., soluble sugars, organic acids) can intensely stimulate microbial activity, even if the C/N ratio is not exceptionally high, triggering the rapid assimilation and immobilization of inorganic nitrogen [29,63,64].

For specific materials such as green waste, nitrogen immobilization is shaped by a unique suite of chemical attributes. First, its exceptionally high C/N ratio (often >100) provides the fundamental prerequisite for intensive nitrogen immobilization [65]. Second, the quality of its carbon (C) pool exhibits a distinct duality. On one hand, it is rich in lignin-derived phenolic compounds [3], which can selectively inhibit or modulate the activity of specific microbial communities through allelochemical effects, thereby regulating microbial nitrogen immobilization processes [66]. On the other hand, green waste is typically abundant in labile carbon fractions (e.g., organic acids) [67], which serve as preferred carbon sources for microbes and stimulate microbial proliferation [68,69]. This coexistence of inhibitory and stimulatory carbon patterns significantly complicates the microbial nitrogen immobilization process. Crucially, phenolics and their oxidation products (e.g., quinones) not only modulate biological processes but also directly participate in abiotic chemical immobilization. They chemically react with inorganic N (e.g., NH_4_^+^) to form stable quinone-ammonium complexes or aromatic organic N, sequestering nitrogen into a chemically stable pool with low turnover rates [65,70]. Consequently, nitrogen immobilization in green waste growing media is the synergistic result of microbial drivers and chemical reactions. Its intensity, dynamics, and the stability of the resulting N pool are jointly determined by the relative concentrations and interactions among labile carbon, recalcitrant lignocellulose, and reactive phenolics. This defines its unique nitrogen immobilization properties—distinct from those of other organic wastes—and serves as the fundamental basis for scientific management and resource valorization of green waste.

### 4.2. Medium Environment

Substrate environmental factors profoundly influence the intensity, pathways, and ultimate fate of nitrogen immobilization by modulating microbial activity and the microenvironmental conditions of chemical reactions. Rather than acting in isolation, these factors synergistically regulate microbial processes, inorganic N availability, mass transfer, and redox microenvironments to jointly shape the dynamic landscape of nitrogen immobilization.

#### 4.2.1. The Role of pH

pH is a key environmental factor regulating both the pathways and intensity of nitrogen immobilization in growing media, affecting both biotic and abiotic processes. In the biotic pathway, pH influences nitrogen transformations by shaping microbial community structure and regulating enzyme activity and functional gene expression [71]. Acidic conditions (pH < 5.5) inhibit nitrification and promote NH_4_^+^ accumulation, thereby promoting microbial NH_4_^+^ assimilation and immobilization [72,73]. However, strongly acidic conditions also suppress organic matter decomposition, limiting energy supply and reducing microbial NO_3_^−^ immobilization [59]. Conversely, neutral to slightly alkaline conditions generally stimulate microbial activity to enhance nitrogen immobilization.

pH is equally critical for abiotic pathways: In the abiotic pathway, pH controls the chemical reactivity of nitrogen species and organic matter. Lignin degradation products (e.g., phenolics) can react with amino acids or NH_3_ via condensation reactions to form brown nitrogenous humic acids, a process facilitated under alkaline conditions [74]. Weakly acidic conditions promote the abiotic adsorption of NH_3_/NH_4_^+^ onto organic matter or clay minerals [75]. Furthermore, lower pH environments promote the generation of NO_2_^−^ and its rapid reaction with reactive organic components like phenolics, forming stable “black nitrogen” compounds [54,76]. This abiotic process can reach completion within minutes without microbial involvement, serving as a vital N-retention mechanism in organic-rich acidic media [48,52]. Collectively, pH regulates the balance between biotic and abiotic N immobilization pathways, playing a pivotal role in regulating N cycling and availability in green-waste-based growing media.

#### 4.2.2. Electrical Conductivity

High electrical conductivity (EC) acts as a physiological stressor that suppresses nitrogen immobilization primarily through osmotic imbalance and ion-specific toxicity [77,78]. These conditions inhibit microbial proliferation and metabolic vigor, effectively lowering the biological demand for inorganic nitrogen assimilation. Beyond direct immobilization, excessive salinity disrupts the kinetics of mineralization and nitrification, leading to an accumulation of unsequestered mineral nitrogen [79,80]. Consequently, the diminished microbial “buffering” capacity under high EC conditions significantly elevates the risk of nitrogen leaching and gaseous losses in green-waste-based media.

#### 4.2.3. Temperature

Temperature regulates nitrogen immobilization by modulating microbial metabolic kinetics and enzymatic activity Within the biological range of 5–25 °C, warming typically accelerates organic matter decomposition; provided that labile carbon is sufficient, this leads to an intensified microbial demand for inorganic nitrogen. However, this stimulatory effect is often self-limiting. Sustained high temperatures can lead to the rapid exhaustion of available carbon sources or increase microbial maintenance energy requirements, eventually shifting the system toward the remineralization of previously immobilized nitrogen [81]. However, this stimulatory effect is subject to a threshold; sustained warming may lead to rapid substrate depletion or increased maintenance and metabolic costs for microorganisms. These factors can weaken long-term nitrogen immobilization capacity and potentially accelerate the remineralization of previously immobilized nitrogen [82]. Overall, temperature primarily regulates the rate and temporal dynamics of nitrogen immobilization, rather than its net accumulation.

#### 4.2.4. Moisture and Oxygen Concentration

Moisture and oxygen concentrations jointly regulate the redox status of the substrate, profoundly influencing the pathways and efficiency of nitrogen immobilization. Insufficient moisture levels limit the diffusion of growing media and nutrients, thereby inhibiting microbial activity. Conversely, excessive moisture leads to pore–water saturation, creating anaerobic microenvironments that suppress aerobic microbial processes and potentially divert N flux toward loss pathways dominated by denitrification [83]. The pore structure and connectivity of the substrate directly determine the uniformity of oxygen transport; moreover, substrate compaction or structural degradation can exacerbate local hypoxia. Therefore, maintaining optimal moisture levels and a well-aerated structure is a critical prerequisite for ensuring adequate oxygen supply, promoting aerobic microbial assimilation-driven nitrogen immobilization, and minimizing nitrogen loss.

Overall, nitrogen immobilization in growing media is governed by the coupled effects of substrate properties and environmental conditions, rather than any single factor Table 1. Substrate composition—particularly C/N ratio and carbon quality—determines the baseline potential and dominant pathways of nitrogen immobilization. Labile carbon generally promotes microbial assimilation, whereas lignin- and phenolic-rich components favor abiotic stabilization and the formation of more persistent nitrogen pools.

Environmental factors regulate the expression of this potential. Among them, pH acts as a key integrator by simultaneously influencing microbial activity, nitrogen speciation, and the chemical reactivity of organic matter. Moisture and oxygen conditions control mass transfer and redox status, thereby determining whether nitrogen is retained through immobilization or lost via processes such as denitrification. Temperature primarily regulates the rate and temporal dynamics of these processes, while elevated salinity (EC) generally suppresses microbial activity and weakens nitrogen retention capacity.

Taken together, nitrogen immobilization can be understood as a system-level process governed by the interaction between intrinsic substrate characteristics and external environmental controls. These interactions ultimately determine its intensity, dominant pathway (microbial vs. abiotic), and implications for nitrogen availability and loss risk in green-waste-based growing media.

**Table 1:** Key factors regulating nitrogen immobilization in growing media.

Influence Factor	Condition	Dominant Pathway	Mechanism
Substrate	Labile C	Microbial	Rapid microbial assimilation
	Recalcitrant C	Abiotic	Chemical stabilization
Nitrogen form	NH_4_^+^-dominated	Microbial/abiotic	Preferentially assimilated by microbes; can be directly adsorbed onto organic matter and clay minerals
	NO_3_^−^-dominated	Microbial	Requires reduction before assimilation
	NO_2_^−^ (intermediate)	Abiotic	Rapidly reacts with phenolic compounds to form stable “black nitrogen”
pH	Acidic	Mixed	Inhibits nitrification; promotes NH_4_^+^ and NO_2_^−^ reactions
	Neutral–alkaline	Mixed	Stimulates microbes and phenolic reactions
Moisture & oxygen	Aerobic	Microbial	Supports microbial activity
	Anaerobic/excessive moisture	Loss pathways	Denitrification
Temperature	5–25 °C	Microbial	Enhances decomposition
	Sustained warming	Microbial	C depletion
EC	High EC	Suppressed microbial	Osmotic stress, ion toxicity

## 5. Strategies for Regulating Nitrogen Immobilization in Growing Media

Based on profound mechanistic insights into nitrogen immobilization and its key drivers, effectively regulating N sequestration during the transformation of green waste into growing media is pivotal for converting this environmental burden into a controllable resource.

### 5.1. Source Pretreatment to Modify Material Properties

Theoretically, a nitrogen pre-loading strategy can satisfy the immobilization demand of the substrate in advance. However, this approach substantially elevates total nitrogen inputs throughout the growing cycle and diminishes nitrogen-use efficiency [84]. Therefore, reducing the intrinsic nitrogen immobilization potential of the substrate at the source represents a more fundamental and efficient solution. The core of source-based pretreatment lies in directly altering the chemical properties of green waste to weaken the internal drivers of nitrogen immobilization. Aerobic composting remains the most conventional and widely used method. It relies on biochemical processes to promote the microbial consumption of labile carbon fractions, leading to the bio-stabilization of green waste. This process also significantly lowers the C/N ratio, thereby reducing the availability of carbon sources for microbial assimilation in the rhizosphere [85]. However, composting is typically time-consuming and may require substantial space, labor, and process control, which can limit its scalability and economic feasibility in large-scale applications. Moreover, incomplete or poorly managed composting may result in residual phytotoxic compounds or unstable organic matter, leading to variable performance in practice. Recently, ammonium salt incubation has emerged as a promising method for rapid detoxification and reduction of nitrogen immobilization potential [67]. Studies have confirmed that this method effectively lowers the nitrogen immobilization capacity of green waste, offering innovative insights for the development of high-efficiency pretreatment technologies. Nevertheless, the long-term stability of this treatment and its potential impacts on substrate salinity and microbial community structure remain insufficiently understood, which may constrain its broader application.

Thermal treatment is currently one of the most widely studied pre-conditioning strategies for wood fiber materials. It reduces the availability of readily degradable components in the fibers through thermal modification and increases the resistance of the material to microbial decomposition, thereby alleviating nitrogen immobilization [86]. Despite its effectiveness, thermal treatment is energy-intensive and may increase production costs, raising concerns regarding its sustainability and large-scale adoption. In addition, excessive thermal modification may negatively affect the physical properties of the substrate, such as porosity and water-holding capacity.

Lignin functionalization represents another, more targeted approach for mitigating nitrogen immobilization [87]. In this method, lignin is first dissolved in an alkaline solution and then mixed with wood fibers. Acid-induced deposition subsequently enables lignin to reattach to both inner and outer fiber surfaces, thereby reconstructing a degradation-resistant barrier similar to that of natural wood. This treatment significantly reduces microbial respiration and nitrogen immobilization in wood fibers. It suggests that limiting rapid microbial utilization of carbon through surface functionalization is an effective strategy for alleviating nitrogen immobilization in wood-fiber-based substrates. However, this approach involves multiple chemical processing steps and may increase operational complexity and cost. Furthermore, its feasibility at commercial scale and potential environmental impacts of chemical reagents require further evaluation.

### 5.2. Process Blending to Optimize Substrate Formulation

During the substrate formulation stage, the scientific blending of additives with diverse properties is intended to actively optimize the physical, chemical, and biological characteristics of the mixed substrate. This helps establish a rhizosphere environment that inhibits excessive nitrogen immobilization while favoring plant growth. For example, mixing pretreated green waste with low-C/N material (e.g., decomposed livestock manure or biogas residue) can rapidly adjust the overall C/N ratio of the blend to a safe range (typically <25). It also directly provides initial available N, thereby alleviating N stress during the crop seedling stage [88]. Simultaneously, incorporating materials with high cation exchange capacity—such as zeolite or biochar—can adsorb and slowly release nutrients like NH_4_^+^, which effectively buffers the sharp decline in available N concentrations triggered by rapid microbial immobilization. Such a multi-dimensional compounding strategy systematically regulates the intensity and pathways of nitrogen immobilization, leading to optimized substrate performance. However, the effectiveness of blending strategies largely depends on the precise selection of material ratios and properties, and the substantial variability in composition among green waste from different sources further increases uncertainty in practical applications.

### 5.3. Intelligent Prediction for Managing Root-Zone Water and Fertilizer

Nitrogen immobilization in green-waste-based growing media is highly dynamic, and its intensity and dominant pathway vary with substrate properties, environmental conditions, and crop growth stage. In this context, intelligent water and fertilizer management, supported by sensor monitoring and model analysis, provides a promising approach for dynamically regulating nitrogen immobilization. To support such predictive management, several modeling approaches have been developed to describe nitrogen mineralization–immobilization dynamics. Classical models are generally based on carbon–nitrogen (C-N) balance principles, where nitrogen release or immobilization is governed by the stoichiometric relationship between substrate composition and microbial demand. For example, first-order decomposition models coupled with microbial C/N requirements simulate nitrogen fluxes as a function of organic matter decomposition and microbial assimilation [60]. More advanced frameworks incorporate microbial carbon use efficiency and enzyme-mediated processes. They emphasize that nitrogen immobilization arises from interactions among carbon availability, microbial metabolism, and environmental drivers [61].

However, despite these advances, existing models remain limited in their applicability to green-waste-based growing media. Most models have been developed for relatively homogeneous soil systems and often assume stable substrate properties. In contrast, green waste is highly heterogeneous in feedstock composition, lignocellulosic structure, and decomposition stage. Furthermore, current modeling frameworks primarily focus on microbial processes and rarely account for abiotic nitrogen immobilization pathways, such as reactions involving lignin-derived phenolics and reactive nitrogen species.

Building on these modeling foundations, the integration of IoT-based sensing technologies enables continuous monitoring of key root-zone variables, including temperature, moisture status, electrical conductivity, and nutrient concentrations. When coupled with nitrogen transformation kinetics, such data can support the development of adaptive water and fertilizer management strategies that are more closely aligned with crop nutrient demand. Nevertheless, nitrogen immobilization in green-waste-based media is jointly regulated by multiple interacting factors, including substrate composition, microbial activity and abiotic reactions. These interactions may vary substantially across feedstocks and cultivation systems. Therefore, predictive management should be based on a mechanistic understanding of substrate properties and nitrogen transformation processes, rather than through the direct application of generalized model parameters. In particular, in systems where microbial and abiotic immobilization coexist, identifying key process indicators and improving model adaptability across different materials and environmental conditions remain critical challenges.

## 6. Future Research Perspectives

The use of green waste as growing media should be considered within the broader context of urban nutrient cycling, the circular economy, and carbon neutrality, rather than being judged only by short-term horticultural performance. Among the many research directions, the top priority is to develop a mechanistic and quantitative understanding of nitrogen immobilization pathways and their relative contributions under different substrate conditions. Future studies should therefore establish clear links between substrate properties, nitrogen transformation processes, and crop responses throughout the entire cultivation cycle.

A major knowledge gap is the lack of quantitative separation between microbial and abiotic nitrogen immobilization, especially in lignin-rich green waste systems. Addressing this issue should be a primary focus, as it underpins predictive regulation and substrate design. In addition, rapid and practical assessment methods are needed to evaluate nitrogen immobilization potential and its temporal dynamics under real production conditions.

Future work should also prioritize the identification of a small set of reliable indicators—such as pH, inorganic nitrogen form, dissolved organic matter composition, and oxygen status—that can effectively predict immobilization intensity. This approach is more useful than examining many variables with similar importance. It will support nitrogen risk assessment and help standardize different types of substrates.

Another key priority is to develop integrated and adaptable predictive models for green-waste-based media. Existing models, mostly developed for soil systems, do not account for substrate heterogeneity or abiotic processes. Future efforts should therefore focus on incorporating both microbial and abiotic pathways into unified modeling frameworks and validating them under commercial production conditions. These models can then be used to test whether process-based precision water and fertilizer management improves the match between nitrogen supply and crop demand, while reducing nitrogen loss.

Overall, future research should move away from descriptive and fragmented approaches and toward hypothesis-driven, quantitatively integrated studies, with a clear focus on the most impactful mechanisms and applications. Only by clarifying the mechanisms behind the “double-edged sword” effect of nitrogen immobilization can green waste be transformed into a stable, controllable, and agronomically valuable growing-media resource.

## Data Availability

No new data were created or analyzed in this study. Data sharing is not applicable to this article.
